# Increase of Meningitis Risk in Stroke Patients in Taiwan

**DOI:** 10.3389/fneur.2018.00116

**Published:** 2018-03-02

**Authors:** Chie-Hong Wang, Tsung-Li Lin, Chih-Hsin Muo, Chen-Huan Lin, Yu-Chuen Huang, Ru-Huei Fu, Woei-Cherng Shyu, Shih-Ping Liu

**Affiliations:** ^1^Graduate Institute of Biomedical Science, China Medical University, Taichung, Taiwan; ^2^Department of Orthopedics, China Medical University Hospital, Taichung, Taiwan; ^3^Management Office for Health Data, China Medical University Hospital, China Medical University, Taichung, Taiwan; ^4^Center for Translational Medicine, China Medical University Hospital, Taichung, Taiwan; ^5^Genetics Center, Department of Medical Research, China Medical University Hospital, Taichung, Taiwan; ^6^School of Chinese Medicine, College of Chinese Medicine, China Medical University, Taichung, Taiwan; ^7^Department of Social Work, Asia University, Taichung, Taiwan

**Keywords:** hemorrhagic stroke, ischemic stroke, blood–brain barrier, meningitis, cerebrovascular accidents

## Abstract

**Background and purpose:**

The blood–brain barrier (BBB) not only provides a physical obstruction but also recruits and activates neutrophils in cases of infection. Hemorrhagic or ischemic stroke reportedly induces the disruption of the BBB. However, few studies have reported a correlation between the incidence of meningitis in patients with a history of stroke. This study tested the hypothesis that patients with a history of stroke may be more vulnerable to meningitis.

**Methods:**

Stroke and age-matched comparison (*n* = 29,436 and 87,951, respectively) cohorts were recruited from the Taiwan National Health Insurance database (2000–2011). Correlations between the two cohorts were evaluated by Cox proportional hazard regression model, Kaplan–Meier curve, and log-rank tests.

**Results:**

The incidence of meningitis was higher in the stroke cohort compared to that in the comparison cohort [hazard ratio (HR), 2.89; 95% confidence interval (CI), 2.23–3.74, *p* < 0.001]. After adjusting for age, sex, and comorbidities, the estimated HR in the stroke cohort was 2.55-fold higher than that in the comparison cohort (CI, 1.94–3.37; *p* < 0.001). Notably, patients who had experienced hemorrhagic stroke had a higher incidence rate of meningitis than those with a history of ischemic stroke, except for patients older than 75 years (incidence rates in hemorrhagic/ischemic stroke patients, 3.14/1.48 in patients younger than 45 years, 1.52/0.41 in 45- to 64-year group, 1.15/0.90 in 65- to 74-year group, 0.74/0.93 in patients older than 75 years). Moreover, stroke patients who had undergone head surgery had the highest meningitis risk (adjusted HR, 8.66; 95% CI, 5.55–13.5; *p* < 0.001) followed by stroke patients who had not undergone head surgery (adjusted HR, 2.11; 95% CI, 1.57–2.82; *p* < 0.001).

**Conclusion:**

Our results indicated that stroke patients have higher risks of meningitis. Compromised BBB integrity in stroke patients may lead to increased vulnerability to infectious pathogens. In summary, our study provided new evidence of the clinical relationship between stroke and meningitis, and our findings suggest the need for precautions to prevent meningitis in stroke patients.

## Introduction

According to the statistics from the World Health Organization, stroke is the second leading cause of death worldwide (1). Meanwhile, cerebrovascular disease (including stroke) was the leading cause of death in Taiwan in 2016 (2). Moreover, poststroke patient management imposes a heavy financial burden on the families as well as the health care system (3, 4). Age, hypertension, diabetes mellitus, obesity, atrial fibrillation, and head injury are significant risk factors for stroke (5, 6).

It has been reported that the risk of stroke is related to several infectious diseases, including meningitis (7). Bacterial meningitis is a significant cause of morbidity and mortality worldwide (8). Attention has been devoted to the neurological complications of meningitis, including stroke and other neurovascular events (9–13). Various studies have also addressed the incidence of stroke in adult patients with meningitis (10–12, 14). Stroke is a serious complication among adult bacterial meningitis patients and is associated with long-term sequelae and also possibly death (15). Previous studies have reported that hemorrhagic and ischemic stroke not only impair neuronal function but also affect the cerebral vasculature, as indicated by the loss of blood–brain barrier (BBB) integrity (16, 17). Brain proteins might leak into the bloodstream, the dural venous sinuses, or the lymph nodes as a result of the compromised BBB in stroke patients (18). BBB dysfunction may cause several neurological diseases, e.g., multiple sclerosis, Alzheimer’s disease, and meningitis (19). To our knowledge, no study has explored the prevalence of meningitis in stroke patients compared to a healthy population; nevertheless, stroke is considered a common complication among meningitis patients.

This study analyzed the medical records of 29,436 stroke patients and 87,951 age-matched controls acquired from Taiwan National Health Insurance (TNHI) database from 2000 to 2011. The risk factors for stroke, including hypertension, diabetes, hyperlipidemia, atrial fibrillation, and head injury were considered. The correlation between meningitis incidences in stroke patients and the comparison group was also assessed.

## Materials and Methods

### Data Source

This retrospective cohort study used data from a Longitudinal Health Insurance Database (LHID) derived from the TNHI program set up by the Taiwan Bureau of National Health Insurance. The TNHI program has a coverage ratio above 99% because all Taiwanese compulsorily join the program. One million insurants were randomly selected from the year 2000 Registry from among beneficiaries in the LHID. The LHID included all inpatient and outpatient medical records for each insurant from 1996 to 2011. Disease was identified in the LHID based on the International Classification of Diseases, ninth Revision, and Clinical Modification (ICD-9-CM). To avoid researchers obtaining information from specific patients, the identification of insurants was re-coded. This study was also approved by the Institutional Review Board of China Medical University Hospital [CMUH104-REC2-115(CR-2)].

### Study Subjects

We collected data 30,019 patients with a new diagnosis of stroke (ICD-9-CM 430–438) at admission between 2000 and 2011 and defined the date of stroke diagnosis at the index date. Stroke patients with meningitis history were excluded (ICD-9-CM 047 and 320–322, *n* = 583). Finally, we selected 29,436 stroke patients as the stroke cohort. The stroke patients were grouped into hemorrhagic stroke (ICD-9-CM 430–432) and ischemic stroke (ICD-9-CM 433–438) based on the first stroke diagnosis. The comparison cohort (*n* = 87,951) was selected among individuals without stroke and meningitis history and was frequency matched for age (5-year strata; for example, 0–4, 5–9, and 10–14 years of age), sex, and index year at an approximately 3:1 rate.

### End Point, Comorbidity, and Operation

All study subjects were followed up from the index date to the date of meningitis occurrence. Those who did not develop meningitis were followed up until the date that they withdrew from the program or the end of 2011. The comorbidities included hypertension (ICD-9-CM 401–405), diabetes (ICD-9-CM 250), hyperlipidemia (ICD-9-CM 272), atrial fibrillation (ICD-9-CM 427.31), and head injury (ICD-9-CM 850–854, 959.01). All comorbidities were defined before the index date. Head surgery, a kind of stroke treatment (ICD-9 operation code 01 and 02), was used to define the severity of stroke in this study. Patients with head surgery treatment were defined as serious stroke and others were defined as mild stroke. Stroke patients with head surgery within 7 days after stroke occurred were defined as head surgery treatment group.

### Statistical Analysis

Chi-square tests were used to assess the significance of the differences in age group, sex, and comorbidity between stroke and comparison cohorts. The incidences of meningitis were calculated for each cohort. Hazard ratios (HRs) and 95% confidence intervals (CIs) of meningitis in the stroke cohort compared with those of the comparison cohort were calculated using Cox proportional hazard regression analysis. The adjusted Cox model was adjusted for age, sex, and all comorbidities (including hypertension, diabetes, hyperlipidemia, atrial fibrillation, and head injury). The association between meningitis and stroke type was also assessed. Because the interaction test between age and stroke showed a significant difference (*p* = 0.03 in adjusted model), we estimated the age-specific risk of meningitis in the adjusted model. In further analyses, we estimated the association between meningitis and head surgery (stroke severity). Kaplan–Meier analysis was used to plot the cumulative incidence of meningitis, and log-rank tests were used to assess the differences in cumulative incidence between the two cohorts. All analyses were performed using SAS 9.4 (SAS Institute Inc., Cary, NC, USA) with a statistical significance level of *p* < 0.05.

## Results

### Characteristics of the Subject Population

A total of 117,387 study subjects (29,436 stroke patients and 87,951 comparison subjects) were included in this study. The mean age was 67.7 ± 14.4 and 66.9 ± 14.3 years in stroke patients and the comparison group, respectively (Table 1). There were 12,431 women (42.23%) and 17,005 men (57.77%) in the stroke group and 37,033 women (42.11%) and 50,918 men (57.89%) in the comparison group (Table 1). Compared to the comparison group, stroke patients had a higher prevalence of hypertension (71.74 vs. 46.75%, *p* < 0.001), diabetes (27.45 vs. 15.75%, *p* < 0.001), hyperlipidemia (31.11 vs. 22.70%, *p* < 0.001), atrial fibrillation (3.83 vs. 1.60%, *p* < 0.001), and head injury (14.89 vs. 7.90%, *p* < 0.001) (Table 1).

**Table 1:** Demographic characteristics and comorbidity between stroke and age-matched comparison cohort in Taiwan.

	Stroke (*N* = 29,436)		Comparison (*N* = 87,951)		
	*n*	%	*n*	%	*p* value^a^
**Age (years)**					0.88
<45	2,049	6.96	6,147	6.99	
45–64	8,908	30.26	26,724	30.39	
65–74	8,171	27.76	24,513	27.87	
75+	10,308	35.02	30,567	34.75	
Mean (SD)	67.7 (14.4)	66.9 (14.3)	

**Gender**					0.71
Women	12,431	42.23	37,033	42.11	
Men	17,005	57.77	50,918	57.89	

**Comorbidity**					
Hypertension	21,116	71.74	41,114	46.75	<0.001
Diabetes	8,081	27.45	13,850	15.75	<0.001
Hyperlipidemia	9,159	31.11	19,964	22.70	<0.001
Atrial fibrillation	1,128	3.83	1,407	1.60	<0.001
Head injury	4,382	14.89	6,950	7.90	<0.001

*^a^p values were evaluated by the Chi-square test*.

### Cumulative Incidences

**Figure 1:**
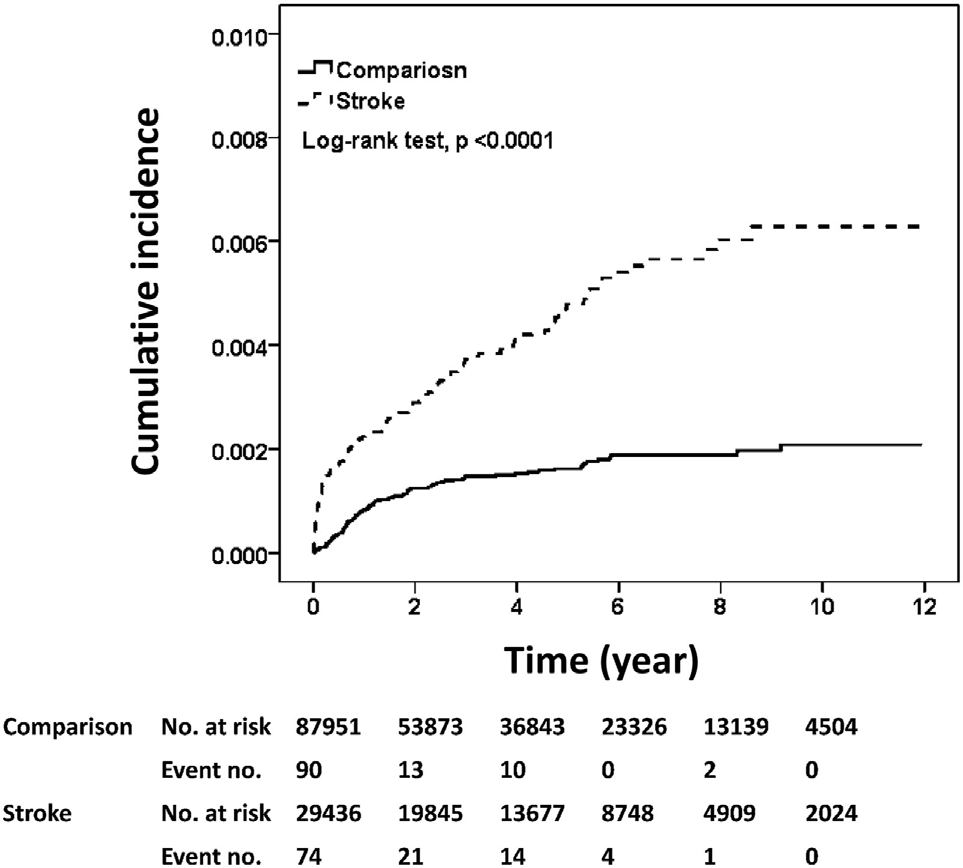
Cumulative incidences of meningitis in the stroke and comparison cohorts. The cumulative incidences of meningitis were plotted according to Kaplan–Meier analysis and the difference in incidences between the two cohorts (stroke cohort, *n* = 29,436; comparison cohort, *n* = 87,951) was evaluated by log-rank test.

The estimated risk of meningitis obtained with the Kaplan–Meier estimator is shown in Figure 1. The cumulative incidence rate of meningitis in the stroke cohort was approximately threefold higher than that in the comparison cohort after a follow-up duration of 12 years (median follow-up, 3.22 years) (0.63 vs. 0.21%, log-rank test, *p* < 0.001).

### Incidence and Risk of Different Meningitis Types between Stroke and Comparison Cohort

We explored the impacts of stroke on developing different types of meningitis (e.g., viral and bacterial meningitis) (Table S1 in Supplementary Material). There was no significant difference for risks of viral meningitis between stroke and comparison cohort (adjusted HR, 0.86; 95% CI, 0.34–2.17). But for bacterial meningitis, stroke patients had a 2.89-fold higher risk compared to comparisons (adjusted HR, 2.89; 95% CI, 2.16–3.88, *p* < 0.001).

### Incidence and Risk of Meningitis According to Stroke Type

Next, we examined the impacts of hemorrhagic and ischemic stroke on meningitis with Cox proportional hazard regression model. As shown by the results summarized in Table 2, 114 of 29,436 stroke patients and 115 of 87,951 comparison group subjects developed meningitis in this study. Our data indicated that stroke was with a significant association for the development of meningitis even after adjusting for age, sex, and comorbidity (crude HR, 2.89; 95% CI, 2.23–3.74, *p* < 0.001; adjusted HR, 2.55; 95% CI, 1.94–3.37, *p* < 0.001). Moreover, the risks of developing meningitis were 4.36- and 2.19-fold higher in hemorrhagic and ischemic stroke patients, respectively, than in the comparison group in the adjusted Cox model (95% CI, 2.91–6.55 and 1.62–2.95, respectively). In addition, the incidence rate of meningitis among hemorrhagic stroke patients (1.65 per 1,000 person-years) was higher than ischemic patients (0.78 per 1,000 person-years) and the comparison group (0.33 per 1,000 person-years) in Cox proportional hazard regression model (Table 2). No significant difference of the duration between index and end point of meningitis occurrence was found between stroke (median, 0.85 year; interquartile range, 2.73 years) and comparison cohort (median, 0.87 year, interquartile range, 1.37 years) (*p* = 0.44, Wilcoxon rank-sum test). The mean duration of meningitis occurrence dates in stroke patients and comparison cohort are 1.79 and 1.52 years, respectively (*p* = 0.30, *t*-test) (Table S2 in Supplementary Material). We also explored the meningitis incidence among different subtypes of stroke (ICD code 430–438). The trend of an increased risk of meningitis in cases vs. controls was consistent across different ICD 9 codes although the estimates for ICD code 433 (occlusion and stenosis of precerebral arteries) and 437 (other and ill-defined cerebrovascular disease) did not reach statistical significance (Table S3 in Supplementary Material).

**Table 2:** Incidence and HR for meningitis in different stroke type.

	*N*	Event no.	Person-years	Rate^a^	Crude HR (95% CI)	Adjusted HR (95% CI)^b^
Comparison	87,951	115	345,336	0.33	1.00	1.00
**Stroke type**						
Overall	29,436	114	125,008	0.91	2.89 (2.23–3.74)***	2.55 (1.94–3.37)***
Hemorrhagic	5,287	32	19,446	1.65	5.24 (3.54–7.75)***	4.36 (2.91–6.55)***
Ischemic	24,149	82	105,562	0.78	2.46 (1.85–3.26)***	2.19 (1.62–2.95)***

*^a^Per 1,000 person-years*.

*^b^Adjusted for age, gender, and comorbidity (including hypertension, diabetes, hyperlipidemia, atrial fibrillation, and head injury)*.

***p < 0.001.

*CI, confidence interval; HR, hazard ratio*.

### Incidence and Risk of Meningitis in Different Stroke Types among Age Groups

The statistics of age-specific incidence and risk of meningitis are shown in Table 3. Stroke patients younger than 45 years age had the highest meningitis risk compared to that in the same age group in the comparison group using Cox model after adjusted age, sex, and comorbidity (adjusted HR, 6.63; 95% CI, 2.98–14.7) as well as the highest incidence rate (2.08 per 1,000 person-years). Meanwhile, the highest prevalence of hemorrhagic stroke occurred in patients younger than 45 years (40.55% in the <45-year group, 23.52% in the 45- to 64-year group, 14.11% in the 65- to 74-year group, and 11.72% in the 75 years or older group). Notably, patients with hemorrhagic stroke histories showed higher meningitis risks and incidence rates compared to those in patients with ischemic stroke histories and the comparison group for each age group except for those 75 years or older. Moreover, our data indicated that stroke patients older than 45 years had a lower risk of developing meningitis (adjusted HR, 1.79 in the 45- to 64-year group, 2.76 in the 65- to 74-year group, 2.12 in the 75 years or older group, and 6.63 in the <45-year group). Collectively, stroke patients older than 45 years only showed an approximately twofold increased risk of meningitis. Stroke patients younger than 45 years with either hemorrhagic or ischemic stroke had increased risks of developing meningitis.

**Table 3:** Incidence and HR for meningitis in different stroke type among age group.

Age (years)	*N*	Event no.	Person-years	Rate^a^	Adjusted HR (95% CI)^b^
**<45**					
Comparison	6,147	10	33,987	0.29	1.00
Stroke type					
Overall	2,049	22	10,589	2.08	6.63 (2.98–14.7)***
Hemorrhagic	831	12	3,820	3.14	9.42 (3.90–22.8)***
Ischemic	1,218	10	6,768	1.48	4.84 (1.92–12.2)***

**45–64**					
Comparison	26,724	40	129,789	0.31	1.00
Stroke type					
Overall	8,908	28	44,794	0.63	1.79 (1.06–3.03)*
Hemorrhagic	2,095	13	8,575	1.52	4.10 (2.13–7.90)***
Ischemic	6,813	15	36,220	0.41	1.18 (0.63–2.22)

**65–74**					
Comparison	24,513	31	95,286	0.33	1.00
Stroke type					
Overall	8,171	35	37,829	0.93	2.76 (1.66–4.59)***
Hemorrhagic	1,153	5	4,360	1.15	3.43 (1.32–8.92)*
Ischemic	7,018	30	33,469	0.90	2.67 (1.57–4.52)***

**75+**					
Comparison	30,567	34	86,274	0.39	1.00
Stroke type					
Overall	10,308	29	31,795	0.91	2.12 (1.27–3.53)**
Hemorrhagic	1,208	2	2,691	0.74	1.71 (0.41–7.14)
Ischemic	9,100	27	29,105	0.93	2.16 (1.28–3.64)**

*^a^Per 1,000 person-years*.

*^b^Adjusted for gender and comorbidity (including hypertension, diabetes, hyperlipidemia, atrial fibrillation, and head injury)*.

*p < 0.05.

**p < 0.01.

***p < 0.001.

*CI, confidence interval; HR, hazard ratio*.

### Incidence and Risk of Meningitis in Different Stroke Types by Head Treatment and Stroke Complication

We evaluated the incidences and HRs for meningitis in stroke patients with or without head treatment, as head surgical procedures were considered the severity of stroke (Table 4). Compared to the comparison group, stroke patients who received head surgery (serious stroke) had a higher risk (HR, 8.66; 95% CI, 5.55–13.5) in Cox model after adjusted for age, sex, and comorbidity, and higher incidence rate (3.33 per 1,000 person-years) of meningitis than stroke patients without head surgery (mild stroke) (HR, 2.11; 95% CI, 1.57–2.82; incidence rate, 0.76 per 1,000 person-years). The same trends were observed in hemorrhagic and ischemic stroke patients compared with those in the comparison group. Patients with hemorrhagic or ischemic stroke histories who underwent head surgery had an over 3.5-fold higher risk of meningitis compared to those without head surgery (3.54-and 5.06-fold in the hemorrhagic and ischemic group, respectively). We also examined the association between meningitis and the stroke complication (pneumonia). Stroke patients still had a higher meningitis risk than comparisons regardless of developing complication or not (adjusted HR, 2.49; 95% CI, 1.88–3.30 in stroke patients without complication, *p* < 0.001; adjusted HR, 4.00; 95% CI, 1.94–8.25 in stroke patients with complication, *p* < 0.001) (Table S4 in Supplementary Material). Nonetheless, there was no significant difference between stroke patients with and without complication in meningitis risk (adjusted HR, 1.61; 95% CI, 0.78–3.32 in stroke patients with complication). Regardless of complication, stroke patients received head surgery treatment had a higher meningitis incidence compared to stroke patients without head surgery and complication (adjusted HR, 3.74; 95% CI, 2.28–6.15 in stroke patients without complication, *p* < 0.001; adjusted HR, 4.66; 95% CI, 1.70–12.8 in stroke patients with complication, *p* < 0.01).

**Table 4:** Incidence and HR for meningitis in different stroke type by head treatment.

	*N*	Event no.	Person-years	Rate^a^	Adjusted HR (95% CI)^b^	
Comparison	87,951	115	345,336	0.33	1.00	
**Overall stroke**
Without head surgery	27,321	89	117,493	0.76	2.11 (1.57–2.82)***	1.00
With head surgery	2,115	25	7,515	3.33	8.66 (5.55–13.5)***	3.84 (2.42–6.11)***

**Hemorrhagic**
Without head surgery	3,453	11	12,826	0.86	2.30 (1.23–4.30)**	1.00
With head surgery	1,834	21	6,620	3.17	8.31 (5.16–13.4)***	3.54 (1.70–7.37)***

**Ischemic**
Without head surgery	23,868	78	104,667	0.75	2.08 (1.54–2.82)***	1.00
With head surgery	281	4	895	4.47	11.2 (4.11–30.5)***	5.06 (1.84–13.9)***

*^a^Per 1,000 person-years*.

*^b^Adjusted for age, gender, and comorbidity (including hypertension, diabetes, hyperlipidemia, atrial fibrillation, and head injury)*.

**p < 0.01.

***p < 0.001.

*CI, confidence interval; HR, hazard ratio*.

### The Prognosis and Outcome of Stroke Patients with or without Meningitis

The mortality rates in stroke patients with and without meningitis development were 50.9 and 42.6%, respectively. Compared to stroke patients without meningitis development, those with meningitis development had a modest increased death risk using Poisson regression after adjusted for age, sex, and comorbidity (relative risk, 1.33; 95% CI, 1.03–1.73, *p* < 0.05) (Table 5).

**Table 5:** Relative risk of death between stroke patients with and without meningitis (*N* = 29,436).

Outcome	*N*	Event no.	%	Relative risk (95% CI)^a^
Death				
Without meningitis	29,322	12,483	42.6	1.00
With meningitis	114	58	50.9	1.33 (1.03–1.73)*

*^a^Adjusted for age, gender, and comorbidity (including hypertension, diabetes, hyperlipidemia, atrial fibrillation, and head injury)*.

*p < 0.05.

## Discussion

Although ischemic stroke is the predominant form of stroke in our study and worldwide, the incidence of meningitis was higher in hemorrhagic stroke patients. The degree of BBB disruption is positively correlated with the severity of intracranial hemorrhage (ICH) (20). We did not find any reports that assessed the association between the severity of ischemic stroke and the degree of BBB damage. However, one study explored the degree of BBB damage in patients with acute ischemic stroke (AIS) who received thrombolytic therapy (21). ICH is the worst complication of intravenous tissue plasminogen activator therapy for AIS. The authors reported that the permeability of the BBB was significantly correlated with the degree of ICH (parenchymal hematoma > hemorrhagic infarction > no hemorrhage). Our findings of a higher meningitis incidence in hemorrhagic stroke patients could be supported by their results. Hemorrhagic stroke patients may have increased risks of meningitis due to the increased permeability of the BBB. One possible underlying mechanism of our study was the upregulation of matrix metalloproteinase-9 (MMP-9) in the brain tissue. Both hemorrhagic and ischemic stroke induced the expression of MMP-9 in the brain tissue; meanwhile, MMP-9 mediated the breakdown of the BBB (22–24). Interestingly, it was reported that the disruption of the BBB in bacterial meningitis was mediated by MMP-9 (25). Since the integrity of the BBB is required for the physical defense against pathogens, higher meningitis risk was observed in stroke patients compared to the comparisons (Table 2). The other group’s study indicated that the plasma concentrations of MMP-9 were significantly higher in either hemorrhagic or ischemic stroke patients than control participants (26). The upregulation of MMP-9 was an early response of stroke; however, we still do not fully understand the impact of MMP-9 on BBB recovery and how the BBB recovers (27). Moreover, another group found microvascular remodeling and disappearances of vasculature close by the injury site of stroke in a long-term *in vivo* investigation (28). These changes may compromise the integrity of BBB permanently. Even though there were no significantly different on the duration between index and end point of meningitis occurrence in stroke patients and comparisons (Table S2 in Supplementary Material), higher meningitis risk was observed in stroke patients compared to the comparisons (Table 2).

Our data showed that stroke patients younger than 45 years had the highest incidence of meningitis. This finding could be explained by our observation that the highest prevalence of hemorrhagic stroke was found in the age group of <45 years. Compared to the other age groups, the meningitis incidence among ischemic stroke patients was slightly higher than that among hemorrhagic stroke patients in those older than 75 years. However, this observation may not be accurate since there were only two meningitis patients with history of hemorrhagic stroke. Since hemorrhagic stroke patients showed a higher meningitis incidence than that in ischemic stroke patients, it was surprising that ischemic stroke patients undergoing head surgery had higher risks of meningitis. The detailed mechanisms should be explored in the future.

### Strengths and Limitations

The major strengths of this study were the large cohort sizes, the long follow-up duration, and the integrated medical records. Nevertheless, this study has several limitations. For example, there were few cases of meningitis patients who suffered hemorrhagic stroke in the 65- to 74-year and older than 75-years age groups. Head surgery, a kind of stroke treatment, was applied to determine the severity of stroke in this study. We did not consider head surgery as a risk factor for meningitis in stroke patients, whereas there was one study exploring the incidence and risk factors of meningitis in patients with craniotomy (29). We did not have the data of MMP-9 expression levels in stroke and comparison cohorts. Due to this limitation, we cannot evaluate the effects of MMP-9 on the development of virus or bacterial meningitis in stroke and comparison cohort. Moreover, we assumed the opportunities for exposure to meningitis pathogens were equal among stroke patients and comparisons in this study. However, it was possibly not the case in the daily life.

## Conclusion

Our data indicated that stroke patients had higher risks of developing meningitis. Patients younger than 45 years with history of hemorrhagic stroke showed the highest risk of meningitis compared to the other groups. Ischemic stroke patients who had undergone head surgery showed an elevated meningitis incidence. In conclusion, our study provides new insight into the clinical relevance of meningitis in stroke patients.

## Author Contributions

C-HW contributed to study design and drafting. C-HM, T-LL, and C-HL contributed to acquisition of data. Y-CH, R-HF, and W-CS contributed to data analysis revising. S-PL contributed to study conception and design, drafting, and revising.

## Conflict of Interest Statement

The authors declare that the research was conducted in the absence of any commercial or financial relationships that could be construed as a potential conflict of interest. The reviewer MG and handling Editor declared their shared affiliation.
