# Supplementary material for: Increase of Meningitis Risk in Stroke Patients in Taiwan
Source: Front Neurol. 2018 Mar 2;9:116. doi: 10.3389/fneur.2018.00116 (PMC5841157; doi:10.3389/fneur.2018.00116)
Supplement: Supplementary file 1 [file table_1.docx]

| Supplementary Table 1. Incidence and hazard ratio for different meningitis types | | | | | |
| --- | --- | --- | --- | --- | --- |
|  | Event no. | Person-years | Rate^†^ | Crude HR (95% CI) | Adjusted HR (95% CI)^‡^ |
| Viral |  |  |  |  |  |
| Comparison | 18 | 345,336 | 0.05 | 1.00 | 1.00 |
| Stroke | 7 | 125,008 | 0.06 | 1.11 (0.46-2.66) | 0.86 (0.34-2.17) |
| Bacterial |  |  |  |  |  |
| Comparison | 97 | 345,336 | 0.28 | 1.00 | 1.00 |
| Stroke | 107 | 125,008 | 0.86 | 3.22 (2.45-4.24)*** | 2.89 (2.16-3.88)*** |
| ^†^per 1000 person-years  ^‡^Adjusted for age, gender and comorbidity (including hypertension, diabetes, hyperlipidemia, atrial fibrillation, and head injury)  *** *p* <0.001 | | | | | |
