# Supplementary material for: Increase of Meningitis Risk in Stroke Patients in Taiwan
Source: Front Neurol. 2018 Mar 2;9:116. doi: 10.3389/fneur.2018.00116 (PMC5841157; doi:10.3389/fneur.2018.00116)
Supplement: Supplementary file 2 [file table_2.docx]

| Supplementary Table 2. The duration between index and end point in subjects with meningitis development | | | | | | | |
| --- | --- | --- | --- | --- | --- | --- | --- |
| Variable | N | Mean | SD | t-test  p-value | Median | IQR | Wilcoxon rank sum test  p-value |
| Comparison | 115 | 1.52 | 1.73 | 0.30 | 0.87 | 1.37 | 0.44 |
| Stroke | 114 | 1.79 | 2.09 |  | 0.85 | 2.73 |  |
|  | | | | | | | |
