# Supplementary material for: Increase of Meningitis Risk in Stroke Patients in Taiwan
Source: Front Neurol. 2018 Mar 2;9:116. doi: 10.3389/fneur.2018.00116 (PMC5841157; doi:10.3389/fneur.2018.00116)
Supplement: Supplementary file 3 [file table_3.docx]

| Supplementary Table 3. Incidence and hazard ratio for meningitis in different stroke type | | | | | | |
| --- | --- | --- | --- | --- | --- | --- |
| Stroke type (ICD-9-CM) | N | Event no. | Person-years | Rate^†^ | Crude HR (95% CI) | Adjusted HR (95% CI)^‡^ |
| Comparison | 87,951 | 115 | 345,336 | 0.33 | 1.00 | 1.00 |
| Stroke type |  |  |  |  |  |  |
| Subarachnoid hemorrhage (430) | 762 | 8 | 2,702 | 2.96 | 9.72 (4.75-19.9)*** | 8.24 (3.97-17.1)*** |
| Intracerebral hemorrhage (431) | 3890 | 20 | 14,488 | 1.38 | 4.40 (2.73-7.07)*** | 3.65 (2.24-5.94)*** |
| Other and unspecified intracranial hemorrhage (432) | 635 | 4 | 2,256 | 1.77 | 5.42 (2.00-14.7)*** | 4.52 (1.66-12.3)** |
| Occlusion and stenosis of precerebral arteries (433) | 924 | 2 | 3,935 | 0.51 | 1.55 (0.38-6.28) | 1.41 (0.35-5.73) |
| Occlusion of cerebral arteries (434) | 12779 | 41 | 53,908 | 0.76 | 2.37 (1.66-3.38)*** | 2.09 (1.44-3.03)*** |
| Transient cerebral ischemia (435) | 3844 | 14 | 19,929 | 0.70 | 2.35 (1.35-4.09)** | 2.13 (1.22-3.74)** |
| Acute, but ill-defined, cerebrovascular disease (436) | 1599 | 6 | 7,707 | 0.78 | 2.64 (1.16-6.00)* | 2.33 (1.02-5.33)* |
| Other and ill-defined cerebrovascular disease (437) | 1581 | 6 | 8,366 | 0.72 | 2.50 (1.10-5.68)* | 2.18 (0.95-4.98) |
| Late effects of cerebrovascular disease (438) | 3422 | 13 | 11,717 | 1.11 | 3.17 (1.78-5.62)*** | 2.76 (1.54-4.96)*** |
| ^†^per 1000 person-years  ^‡^Adjusted for age, gender and comorbidity  *** *p* <0.001 | | | | | | |
