# Supplementary material for: Increase of Meningitis Risk in Stroke Patients in Taiwan
Source: Front Neurol. 2018 Mar 2;9:116. doi: 10.3389/fneur.2018.00116 (PMC5841157; doi:10.3389/fneur.2018.00116)
Supplement: Supplementary file 4 [file table_4.docx]

| Supplementary Table 4. Incidence and hazard ratio for meningitis between complication and head surgery | | | | | | |
| --- | --- | --- | --- | --- | --- | --- |
|  | N | Event no. | Person-years | Rate^†^ | Adjusted HR (95% CI)^‡^ | |
| Comparison | 87,951 | 115 | 345,336 | 0.33 | 1.00 |  |
| Stroke |  |  |  |  |  |  |
| Without complication | 27,492 | 106 | 120,501 | 0.88 | 2.49 (1.88-3.30)*** | 1.00 |
| With complication | 1,944 | 8 | 4,507 | 1.77 | 4.00 (1.94-8.25)*** | 1.61 (0.78-3.32) |
| Comparison | 87,951 | 115 | 345,336 | 0.33 | 1.00 |  |
| Stroke |  |  |  |  |  |  |
| Without complication / without head surgery | 25,674 | 85 | 113,888 | 0.75 | 2.10 (1.56-2.82)*** | 1.00 |
| With complication / without head surgery | 1,647 | 4 | 3,605 | 1.11 | 2.43 (0.89-6.64) | 1.17 (0.43-3.21) |
| Without complication / with head surgery | 1,818 | 21 | 6,613 | 3.18 | 8.41 (5.22-13.5)*** | 3.74 (2.28-6.15)*** |
| With complication / with head surgery | 297 | 4 | 902 | 4.44 | 10.2 (3.76-27.9)*** | 4.66 (1.70-12.8)** |
| ^†^per 1000 person-years  ^‡^Adjusted for age, gender and comorbidity (including hypertension, diabetes, hyperlipidemia, atrial fibrillation, and head injury)  ** *p* < 0.01, *** *p* <0.001 | | | | | | |
